# A systematic review and meta-analysis of the effects of exercise training on dysfunction in acute, subacute, and chronic stroke patients

**DOI:** 10.3389/fneur.2026.1740742

**Published:** 2026-05-14

**Authors:** Jing Tan, Yongyan Tang, Daobin Han

**Affiliations:** 1 Department of Rehabilitation Medicine, Central Hospital of Guangdong Provincial Nongken, Zhanjiang, China; 2 Department of Ultrasonic Diagnosis, The Third People’s Hospital of Jinan, Jinan, China; 3 Medical Laboratory Center, The Second Hospital of Shandong University, Jinan, China

**Keywords:** acute stage, chronic stage, exercise training, meta-analysis, stroke, subacute stage

## Abstract

**Objective:**

This systematic review and meta-analysis aimed to compare the effectiveness of exercise training initiated in the acute, subacute, and chronic phases after stroke.

**Methods:**

The Wanfang, CNKI, VIP, PubMed, Web of Science, Cochrane Library, and EMBASE databases were comprehensively searched for relevant articles from their inception to December 2025. Studies comparing outcomes among the acute stage exercise training group (ASETG), subacute stage exercise training group (SSETG), and chronic stage exercise training group (CSETG) for stroke patients were included. The outcomes assessed were improvements in the Fugl-Meyer Assessment (FMA), Berg Balance Scale (BBS), Action Research Arm Test (ARAT), Modified Barthel Index (MBI), and Modified Ashworth Scale (MAS) after treatment. Meta-analysis of comparable data was performed using Review Manager 5.3 software provided by the Cochrane Collaboration.

**Results:**

A total of 16 randomized controlled trials (RCTs) and 22 observational studies involving 5,254 patients were included. The meta-analysis indicated that the ASETG showed significantly greater improvements in FMA scores compared to the SSETG [WMD = 7.95, 95% CI (6.73, 9.16)]. This trend was also observed in the FMA-UE [WMD = 4.26, 95% CI (3.09, 5.44)] and FMA-LE [WMD = 4.57, 95% CI (3.73, 5.41)] subscales when compared to the SSETG. Furthermore, the ASETG outperformed the CSETG in FMA scores [WMD = 5.31, 95% CI (3.89, 6.72)]. In addition, the ASETG exhibited significantly greater improvements in BBS scores compared to the SSETG [WMD = 3.64, 95% CI (1.14, 6.15)] and in MBI scores [WMD = 10.66, 95% CI (9.55, 11.77)]. Moreover, the SSETG showed a greater improvement in ARAT scores compared to the CSETG [WMD = 2.70, 95% CI (1.81, 3.59)].

**Conclusion:**

This review suggests earlier post-stroke exercise may improve functional outcomes (FMA, BBS, MBI, ARAT). However, due to heterogeneity and inclusion of observational studies, findings should be interpreted cautiously. High-quality RCTs are needed to confirm the optimal timing.

**Systematic review registration:**

https://www.crd.york.ac.uk/PROSPERO/view/CRD42024519257, identifier PROSPERO (CRD42024519257).

## 1 Introduction

Stroke is a cerebrovascular disease caused by damage to brain tissue due to the rupture or blockage of blood vessels in the brain (1). It is typically classified into two categories: hemorrhagic and ischemic stroke. Stroke is the leading cause of death and disability, leading to dysfunctions in cognition, speech, swallowing, movement, sensation, emotion, balance, as well as bowel and bladder control, severely impacting patients’ quality of life (1). Modern rehabilitation theory and practice have demonstrated that regular, effective, and comprehensive rehabilitation treatment after stroke can reduce and improve dysfunction, enhance patients’ self-care ability, prevent disuse, misuse, and overuse syndromes, and avoid complications such as muscle atrophy, joint spasm, shoulder-hand syndrome, and shoulder subluxation (2). Current studies on exercise training at different stages of stroke have confirmed that this intervention benefits recovery of sensorimotor function, cognition, balance, gait, and activities of daily living (ADL) in patients in the acute (3–5), subacute (6–8), and chronic (9, 10) stages of stroke. However, whether differences exist in efficacy between stages remains unclear. According to clinical guidelines, the acute stage is defined as within 48 h to 14 days after stroke onset, the subacute stage as 14 days to 1 month, and the chronic stage as beyond 1 month (11, 12). The commonly used exercise training methods include walking training (3), treadmill training (4, 5, 9), robot-assisted gait training (8, 13), upper limb task-oriented training (14–16), upper limb robot training (17), and balance bed training. A clinical trial reported that exercise training using a non-invasive portable multi-frequency bioimpedance device can significantly increase muscle mass and improve gait abnormalities in the acute phase of ischemic stroke (3). Anna M et al. observed that intensive treadmill training positively impacted sensorimotor function and self-care ability in 20 acute ischemic stroke patients (4). Zhang et al. found that robot-assisted gait training in the subacute stage significantly improved certain spatiotemporal parameters and optimized lower limb joint movement and muscle activation patterns during walking, effects not achieved by conventional gait training (6, 17). Furthermore, high-intensity arm training (7) and robot-assisted hand function training (8) in the subacute stage showed greater improvements in upper limb function compared to the control group. Macko et al. (9) reported that task-oriented aerobic exercise training significantly improved gait abnormalities and ADL in stroke patients during the chronic phase. Ken-Wei Chang et al. found that treadmill-based exercise training effectively improved balance and walking speed compared to pre-exercise levels (10).

We urgently need a series of studies to evaluate which phase—acute, subacute, or chronic—is more effective for rehabilitation therapy in stroke patients (13–50). Studies indicate that exercise training impacts sensorimotor function, gait, balance, and ADL in patients at different stages of stroke. Some studies suggest that early rehabilitation is not always better. Premature rehabilitation can increase oxygen consumption, activate the inflammatory cascade, promote apoptosis, inhibit dendrite growth, affect nerve remodeling, and potentially lead to stroke progression (51, 52). There is ongoing debate about the optimal time for exercise training intervention in stroke rehabilitation. Additionally, few studies have focused on the best timing for exercise training to treat stroke-related dysfunction. Xie et al. (30) conducted a randomized controlled trial exploring the optimal period for hemiplegia training post-stroke. Among 36 participants, improvements in FMA, BBS, and MBI were significantly greater in the acute stage compared to the subacute and chronic stages. However, there were no significant improvements in trunk impairment scale (TIS), MAS, or mini-mental state examination (MMSE). Li et al. (29) found that exercise training using the “Balance-Bed” significantly improved functional independence measure (FIM) and BBS in the acute stage, but no significant differences were observed in the subacute stage. Another randomized controlled trial demonstrated that arm-hand boost therapy in the acute stage produced greater improvements in FMA-UE, ARAT, Jebsen Taylor hand function test (JEBSEN), and Rivermead motor assessment-arm subscale (RMA-A) compared to the chronic stage (15). However, no significant differences were found in stroke upper limb capacity scale (SULCS) or the box and block test (BBT). Johanna et al. (16) reported that functional electrical stimulation produced more significant improvements in FMA-UE during the subacute stage compared to the chronic stage, though no differences were observed in ARAT. In contrast, a randomized controlled trial with 23 participants found that transcranial direct current stimulation and unilateral robot therapy significantly improved FMA, ARAT, motor activity log-28 (MAL), and stroke impact scale (SIS) in the subacute stage, but not in the chronic stage (17). However, a randomized controlled trial involving 53 participants by Alexander et al. showed no significant differences in the effect of upper limb task-oriented training on ARAT across the acute, subacute, and chronic stages of stroke (14). It is clear that determining the optimal timing for exercise training intervention to maximize benefits on sensorimotor function, cognition, balance, gait, and ADL performance across different stroke stages is crucial. However, no systematic reviews or meta-analyses have yet been conducted on the effects of early exercise training for stroke dysfunction. Therefore, there is an urgent need for a systematic review and meta-analysis to determine the optimal timing for exercise training interventions in stroke rehabilitation. This study aims to compare the effectiveness of exercise training at acute, subacute, and chronic stages in treating stroke dysfunction and identify the best intervention timing by evaluating FMA, BBS, MBI, MAS, and ARAT.

## 2 Materials and methods

### 2.1 Search strategy

This study was designed and conducted following the Preferred Reporting Items for Systematic Reviews and Meta-Analyses (PRISMA) guidelines (53). Two reviewers independently performed electronic searches of the Wanfang, CNKI, VIP, PubMed, Web of Science, EMBASE, and Cochrane Library databases up to December 2025. The search terms included “Exercise,” “Physical Activity,” “Physical Exercise,” “Isometric Exercise,” “Aerobic Exercise,” “Exercise Training,” “Training,” “Acute Stage,” “Early Stage,” “Subacute Stage,” “Chronic Stage,” “Convalescence,” “Recovery Phase,” “Restoration Stage,” “Sequela Period,” “Stroke,” “Cerebrovascular Accident,” “Brain Infarction,” “Cerebral Hemorrhage,” “Intracerebral Hemorrhage,” “Hematencephalon,” “Brain Haemorrhage,” “Randomized Controlled Trial,” and “Placebo” (Supplementary Table S1). Additionally, we reviewed reference lists of relevant studies to identify further studies. Any disagreements were resolved by a third author. The search was not restricted by language or publication date. The systematic review and meta-analysis were registered on the PROSPERO website under the registration number CRD42024519257.

### 2.2 Inclusion and exclusion criteria

We included randomized clinical trials (RCTs) and observational studies that met the following criteria: (1) patients with stroke were older than 18 years; (2) intervention measures included exercise training such as walking, treadmill, robot-assisted gait training, upper limb task-oriented training, upper limb robot training, and balance bed training; (3) at least one outcome was reported and used for meta-analysis; (4) comparison of outcomes among the acute, subacute, and chronic stages was provided; and (5) the full text was available. Studies were excluded if: (1) patients were younger than 18 years; (2) they were reviews, case reports, duplicates, or conference abstracts; (3) they were basic research involving animals or cadavers; (4) they were non-controlled studies; or (5) we were unable to obtain the full text or accurate data. Additionally, patients with severe heart disease, respiratory disease, postural hypotension, musculoskeletal injury, severe osteoporosis, or other conditions intolerant of exercise training were excluded.

### 2.3 Literature review

Two researchers independently searched for literature using the keywords and managed the references using Endnote software to remove duplicates and extract relevant information from the titles and abstracts. After excluding studies based on the criteria, the full texts were reviewed to determine eligibility. Discrepancies were resolved through discussion or by a third senior researcher.

### 2.4 Data extraction

Data were extracted using a pre-designed table. Two independent researchers extracted the following information: first author, publication date, country, study design, sample size, mean age, gender, type of exercise training, timing of intervention, follow-up period, and relevant clinical outcomes. Regarding rehabilitation dose, the classification was informed by the European Stroke Organization (ESO) 2025 guideline on motor rehabilitation, which recommends a minimum of 20 additional hours of repetitive task-specific training beyond usual care to improve upper limb and walking function. To explore the dose–response relationship, we categorized the included studies based on the total therapy hours per complete rehabilitation cycle reported in each study. Specifically, studies with <1,000 h were classified as the low-dose group (representing interventions that exceed the ESO-recommended dose but are at the lower end of the distribution in this meta-analysis), studies with 1,000–2,000 h as the medium-dose group (reflecting long-term, high-intensity rehabilitation regimens), and studies with >2,000 h as the high-dose group (capturing the maximal effects of cumulative, multi-cycle, or extended rehabilitation) (54).

### 2.5 Outcome measures

We included studies that reported at least one of the following outcomes: improvement in FMA, BBS, MBI, MAS, and ARAT after treatment.

### 2.6 Quality assessment

Two researchers independently assessed the quality of the RCTs and observational studies, with disagreements resolved through discussion or by a third senior researcher. The Cochrane Risk of Bias (ROB) assessment tool was used to evaluate the quality of each RCT (55). The risk of bias was assessed in several areas: random sequence generation, allocation concealment, blinding of participants and personnel, blinding of outcome assessment, incomplete outcomes, and selective reporting. Each entry was rated as “Yes” (low risk of bias), “No” (high risk of bias), or “Unclear” (insufficient information or uncertainty regarding potential bias) (55). If all items were rated “Yes,” the study received an “A” grade for high quality. If one or more items were marked as “Unclear,” the study was rated as “B” (moderate quality). If any item was rated as “No,” the study was graded as “C” (low quality). The Newcastle Ottawa Scale (NOS) (56) was used to evaluate the quality of observational studies, assessing bias across three domains: selection of study subjects (4 points), comparability of groups (2 points), and ascertainment of exposure or outcome (3 points). The maximum score was 9, with higher scores indicating better quality. Studies were classified as high, moderate, or low quality with scores of ≥7, 4–6, and ≤3, respectively.

The certainty of evidence for each outcome was assessed using the Grading of Recommendations Assessment, Development and Evaluation (GRADE) framework. Two independent reviewers evaluated the quality of evidence for each outcome based on five domains: risk of bias (study limitations), inconsistency, indirectness, imprecision, and publication bias. The certainty of evidence was classified into four levels: high, moderate, low, or very low. Any disagreements were resolved through discussion or by consulting a third reviewer. The GRADE evidence quality assessment are shown in Table 1.

**Table 1:** GRADE evidence quality assessment.

Certainty assessment							No. of patients		Effect		Certainty	Importance
No. of studies	Study design	Risk of bias	Inconsistency	Indirectness	Imprecision	Other considerations	(Interference)	(Comparison)	Relative (95% CI)	Absolute (95% CI)		
Exercise training for FMA improvement: acute phase versus subacute phase												
7	Randomized trials	Serious^a^	Not serious	Not serious	Not serious	None	257	261	–	MD 7.95 higher (6.73 higher to 9.16 higher)	⨁⨁⨁◯ Moderate^a^	
Exercise training for FMA improvement: acute phase versus chronic phase												
8	Randomized trials	Serious^b^	Not serious	Not serious	Not serious	None	335	334	–	MD 5.31 higher (3.89 higher to 6.72 higher)	⨁⨁⨁◯ Moderate^b^	
Exercise training for FMA improvement: subacute phase versus chronic phase												
5	Randomized trials	Not serious	Not serious	Not serious	Serious^c^	None	120	114	–	MD 0.24 higher (1.34 lower to 1.81 higher)	⨁⨁⨁◯ Moderate^c^	
Exercise training for FMA-UE improvement: acute phase versus subacute phase												
8	Randomized trials	Serious^d^	Not serious	Not serious	Not serious	None	440	426	–	MD 4.26 higher (3.09 higher to 5.44 higher)	⨁⨁⨁◯ Moderate^d^	
Exercise training for FMA-LE improvement: acute phase versus subacute phase												
7	Non-randomized studies	Not serious	Not serious	Not serious	Not serious	None	430	418	–	MD 4.57 higher (3.73 higher to 5.41 higher)	⨁⨁◯◯ Low	
Exercise training for BBS improvement: acute phase versus subacute phase												
3	Non-randomized studies	Not serious	Not serious	Not serious	Serious^e^	None	48	47	–	MD 3.64 higher (1.14 higher to 6.15 higher)	⨁◯◯◯ Very low^e^	
Exercise training for MBI improvement: acute phase versus subacute phase												
18	Randomized trials	Serious^f^	Not serious	Not serious	Not serious	None	1,876	1,368	–	MD 10.66 higher (9.55 higher to 11.77 higher)	⨁⨁⨁◯ Moderate^f^	
Exercise training for MBI improvement: subacute phase versus chronic phase												
9	Randomized trials	Serious^g^	Very serious^h^	Not serious	Not serious	None	800	704	–	MD 2.88 higher (1.05 higher to 4.7 higher)	⨁◯◯◯ Very low^g,h^	
Exercise training for MAS improvement: subacute phase versus chronic phase												
2	Randomized trials	Not serious	Not serious	Not serious	Very serious^i^	None	24	18	–	MD 0.26 higher (0.09 lower to 0.62 higher)	⨁⨁◯◯ Low^i^	
Exercise training for ARAT improvement: subacute phase versus chronic phase												
5	Randomized trials	Not serious	Not serious	Not serious	Serious^j^	None	117	110	–	MD 2.7 higher (1.81 higher to 3.59 higher)	⨁⨁⨁◯ Moderate^j^	

CI, confidence interval; MD, mean difference. ^a^Assessment of randomization. ^b^Allocation concealment. ^c^The total sample size for continuous outcomes is less than 400. ^d^Blinding. ^e^The total sample size for continuous outcomes is less than 400. ^f^Blinding. ^g^Selective reporting. ^h^I^2^ > 75%. ^i^The sample size was very small. ^j^The total sample size for continuous outcomes is less than 400.

### 2.7 Data synthesis and analysis

The Review Manager 5.3 software provided by the Cochrane Collaboration Network was used to perform the meta-analysis of comparable data. Odds ratios (ORs) with 95% confidence intervals (CI) were calculated for dichotomous outcomes, while weighted mean differences (WMDs) with 95% CI were used for continuous data. Heterogeneity among studies was tested using chi-square (χ^2^) and I-square (I^2^) tests. A *p*-value >0.10 and *I*^2^ ≤ 50% were considered to indicate insignificant heterogeneity. A fixed-effect model was applied in cases of insignificant heterogeneity (*p* > 0.10, *I*^2^ ≤ 50%), while a random-effect model was used when significant heterogeneity existed (*p* < 0.10, *I*^2^ > 50%). Sensitivity analyses were conducted to explore the sources of heterogeneity by omitting one study at a time and pooling the remaining data to determine the stability of the outcomes. Egger’s test or funnel plots were employed to assess publication bias when 10 or more studies were included (55). Funnel plots were drawn using Review Manager 5.3, and Egger’s test was conducted in STATA 12 with a significance level of *p* < 0.05.

## 3 Results

### 3.1 Study selection and characteristics

**Figure 1:**
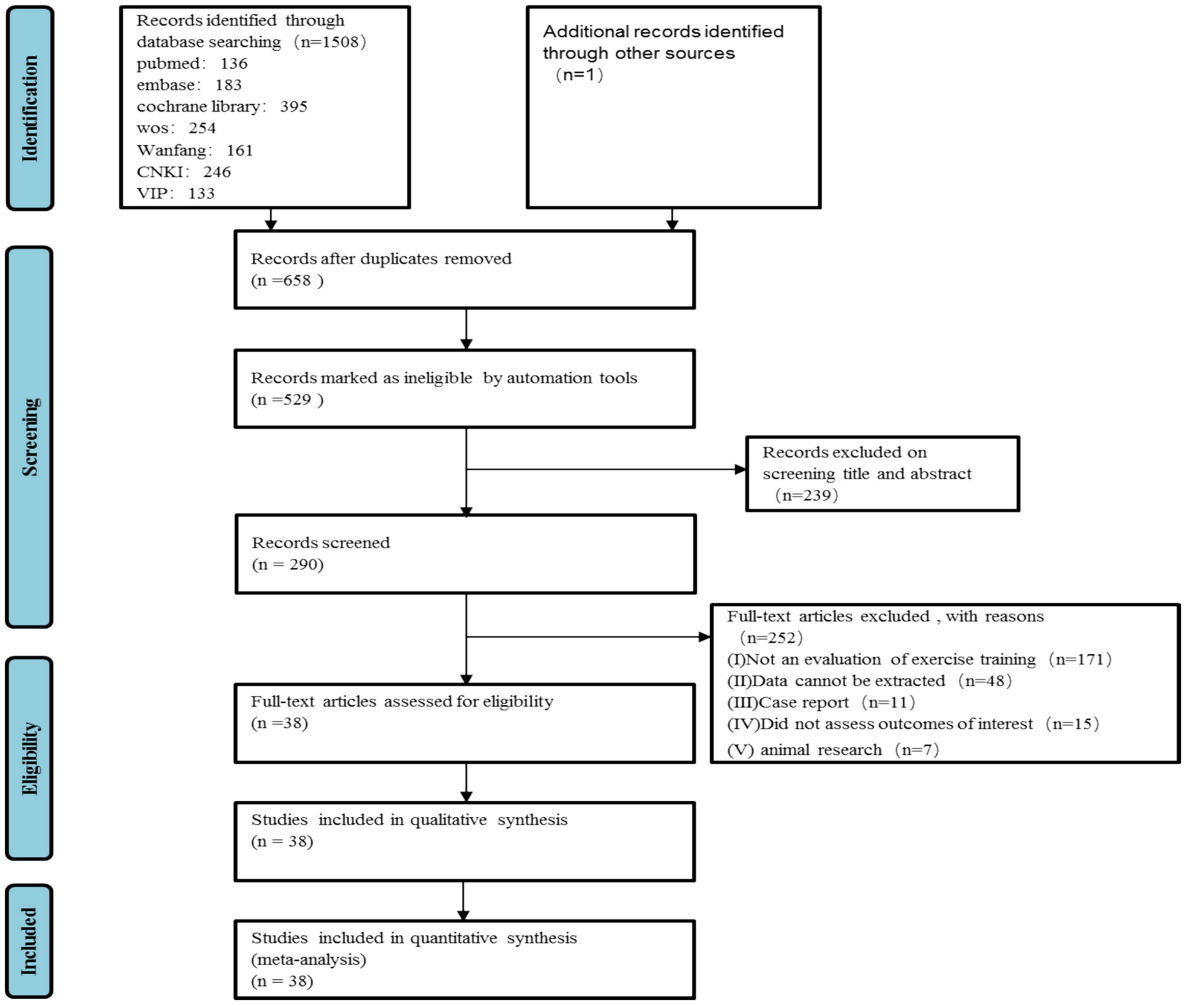
PRISMA flow diagram of study selection.

A total of 1,508 articles were initially identified. After removing 851 duplicates using Endnote Document Management software, 129 articles were excluded by automation tools, and 239 were excluded after reviewing titles and abstracts for failing to meet the inclusion criteria. Following full-text review and quality assessment, 252 unqualified articles were further excluded. One additional article was manually retrieved from the reference lists of relevant reviews. Ultimately, 38 articles met the eligibility criteria and were included in the meta-analysis (13–50). Of these, 16 were RCTs (13–28), and 22 were observational studies (29–50). The PRISMA flow diagram illustrating the study selection process is shown in Figure 1.

A total of 5,254 participants were included across the 38 studies (13–50). Thirty-one studies involved participants in the acute stroke phase, 32 studies included participants in the subacute stroke phase, and 22 studies included participants in the chronic stroke phase. The basic features of the included research are shown in Table 2.

**Table 2:** General characteristics.

Reference	Subjects number			Age (years)			Start time of rehabilitation			Time of assessment	Indicators of evaluation
	ASETG	SSETG	CSETG	ASETG	SSETG	CSETG	ASETG	SSETG	CSETG		
Park et al. (13)	9	8	–	52.22 ± 15.89	53.13 ± 12.00	–	<42 days	>42 days	–	After 64 days	⑤⑥
Martinuzzi et al. (14)	10	8	–	64.05 ± 2.29	68.42 ± 2.29	–	<14 days	>14 days	–	After 28 days	②⑦
Meyer et al. (15)	–	19	13	–	68.30 ± 7.21	67.71 ± 7.21	–	>14 days	>30 days	After 28 days	②⑦
Dromerick et al. (16)	16	17	20	61.80 ± 11.30	63.90 ± 10.80	67.30 ± 9.80	<30 days	30–90 days	90–180 days	After 42 days	⑦
Triccas et a. (17)	–	12	11	–	67.16 ± 10.10	62.63 ± 9.30	–	>14 days	>30 days	After 56 days	①⑦
Cramer et al. (18)	–	43	40	–	60.20 ± 11.40	60.80 ± 10.90	–	28–84 days	>84 days	After 30 days	④
Wang et al. (19)	31	31	–	61.20 ± 8.40	60.80 ± 9.10	–	1–7days	>7 days	–	After 30 days	①⑥
Patel et al. (20)	–	26	26	–	60.20 ± 11.40	60.80 ± 10.90	–	14–30 days	>30 days	After 30 days	②⑤⑦
Mancuso et al. (21)	192	67	32	70.20 ± 10.40	70.90 ± 11.00	70.00 ± 9.90	6–19 days	20–37 days	>37 days	After 42 days	①⑤
Musicco et al. (22)	779	489	448	69.50 ± 10.40	69.20 ± 10.10	69.27 ± 9.80	<14 days	15–30 days	>30 days	After 30 days	②⑦
Sahoo and Nehal (23)	19	8	–	56.30 ± 13.54	58.25 ± 12.90	–	<30 days	30–150 days	–	After 14 days	⑤
Lai et al. (24)	123	38	–	70.80 ± 10.50	73.50 ± 11.00	–	<1 day	3–4 days	–	After 30 days	⑤
Suqin et al. (25)	40	40	–	64.65 ± 11.62	64.55 ± 11.64	–	<1 day	>3 days	–	After 14 days	⑤⑥
Ruisen and Shujie (26)	32	32	–	60.78 ± 3.48	61.03 ± 3.20	–	<14 days	<28 days	–	After 28 days	⑤
Shuhong et al. (27)	90	90	–	71.48 ± 3.70	71.50 ± 3.69	–	<14 days	14–28 days	–	After 28 days	②③⑤
Qunfeng et al. (28)	54	–	54	64.68 ± 7.03	–	64.25 ± 6.96	<14 days	–	>30 days	After 28 days	①⑤
Oddsson et al. (29)	7	2	–	67.00 ± 12.51	71.15 ± 11.36	–	<14 days	>14 days	–	After 28 days	④
Xie et al. (30)	11	15	10	63.18 ± 12.61	68.33 ± 11.29	68.43 ± 9.43	<7 days	14–168 days	>168 days	After 12 days	①④⑤⑥
Mingyuan and Cuihong (31)	108	108	–	–	–	–	<14 days	>14 days	–	After 28 days	②③⑤
Feng et al. (32)	19	–	54	52.30 ± 16.10	–	50.80 ± 12.80	<30 days	–	>30 days	After 48 days	⑤
Jun and Fanglian (33)	40	40	–	57.80 ± 8.80	68.10 ± 8.90	–	2–7 days	21–28 days	–	After 28 days	⑤
Kun and Pengcheng (34)	50	50	–	–	–	–	2–14 days	15–30 days	–	After 28 days	⑤
Yali et al. (35)	–	80	80	–	56.00 ± 9.40	57.20 ± 9.60	–	14–28 days	>30 days	After 14 days	⑤
Huasheng and Chunlan (36)	40	36	–	60.70 ± 9.80	62.10 ± 8.60	–	2–7 days	21–28 days	–	After 28 days	②③⑤
Qinglan et al. (37)	30	30	30	56.83 ± 10.89	57.83 ± 9.54	57.97 ± 9.16	<14 days	15–90 days	>90 days	After 30 days	⑤
Xing (38)	30	30	30	62.12 ± 9.84	60.29 ± 8.42	58.97 ± 7.61	<14 days	14–28 days	>30 days	After 84 days	①④⑤
Yanfang (39)	32	–	32	65. 40 ± 3.30	–	64.40 ± 3.80	<14 days	–	>30 days	After 30 days	①⑤
Hong (40)	–	48	48	–	55.28 ± 2.36	55.35 ± 2.40	–	>14 days	>30 days	After 90 days	②③⑤
Chao (41)	109	–	109	65.40 ± 1.40	–	65.20 ± 1.20	<14 days	–	>30 days	After 42 days	①⑤
Xiaofei (42)	50	–	50	66.21 ± 6.37	–	66.82 ± 6.41	<14 days	–	>30 days	After 30 days	②③⑤
Lanwen (43)	33	33	33	63.50 ± 14.60	63.60 ± 14.80	63.70 ± 14.90	<3 days	3–14 days	>30 days	After 42 days	①
Zongjun (44)	30	30	30	63.50 ± 3.50	65.40 ± 2.90	67.80 ± 4.60	<3 days	3–14 days	>30 days	After 42 days	①
Yuanqiu (45)	22	22	22	68.30 ± 5.60	70.10 ± 3.20	66.20 ± 6.70	<3 days	3–14 days	>30 days	After 42 days	②③
Junyanand and Guangyu (46)	36	–	36	60.00 ± 5.60	–	60.00 ± 5.60	>2 days	–	>30 days	After 42 days	①⑤
Lianjin et al. (47)	60	60	–	–	–	–	≤15 days	≥15 days	–	After 30 days	⑤
Qingrong et al. (48)	72	64	–	–	–	–	2–14 days	15–30 days	–	After 30 days	②③⑤
Dongjun et al. (49)	108	108	–	–	–	–	2–14 days	15–30 days	–	After 30 days	②③⑤
Ying et al. (50)	40	40	–	62.40 ± 8.70	70.10 ± 4.70	–	2–7 days	21–28 days	–	After 30 days	②③⑤

① Fugl-Meyer Assessment Scale(FMA), ② Upper Extremity Fugl-Meyer Assessment(FMA-UE), ③ Lower extremity Fugl-Meyer assessment (FMA-LE), ④ Berg Balance Scale(BBS), ⑤ Modified Barthel Index (MBI), ⑥ Modified Ashworth Scale (MAS), ⑦ Action Research Arm Test(ARAT).

**Figure 2:**
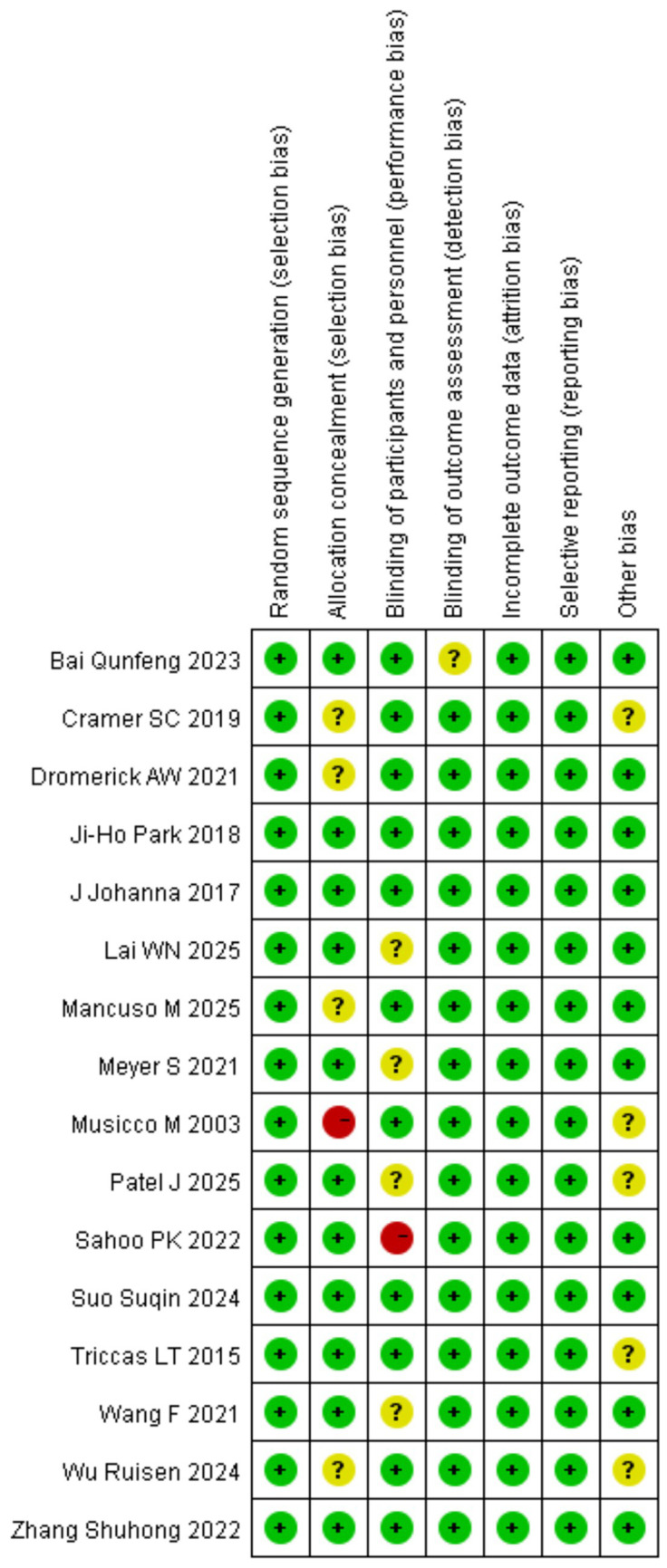
Risk of bias assessment of randomized controlled trials.

**Figure 3:**
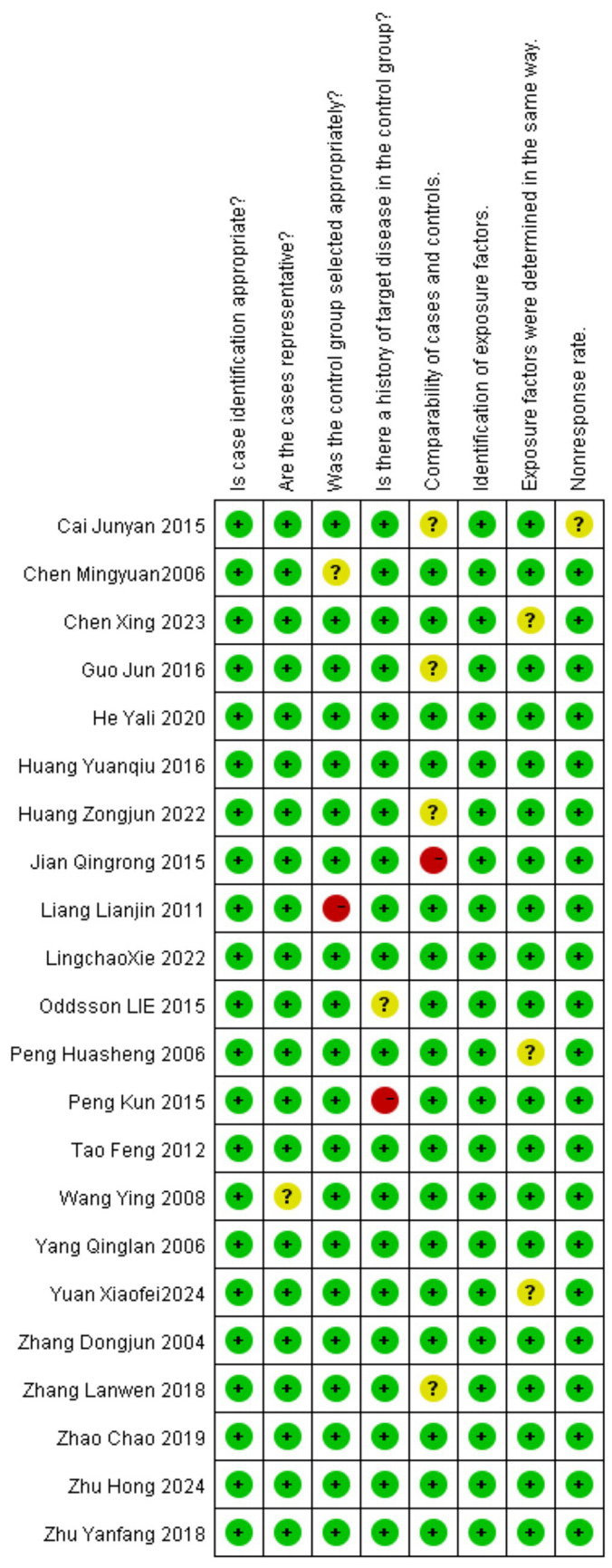
Risk of bias assessment of observational studies.

The Cochrane Collaboration’s tool was used to assess the risk of bias in RCTs (55). Four studies received a quality grade of “A,” 10 were graded “B,” and two was graded “C.” According to the Newcastle-Ottawa scale (NOS) (56), nine study was rated as high quality (total score ≥7), and 13 study was rated as moderate quality (total score between 4 and 6). The summary of risk of bias scores for the RCTs and observational studies is presented in Figures 2, 3.

### 3.2 Meta-analysis results

#### 3.2.1 Fugl-Meyer Assessment improvement after treatment

**Figure 4:**
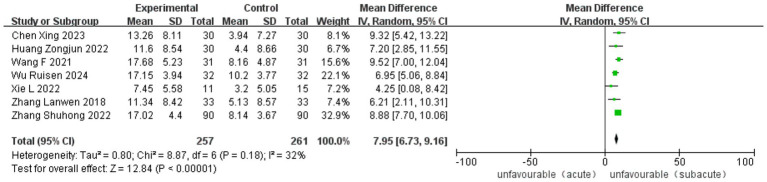
Forest plot for comparison of length of FMA between ASETG and SSETG.

**Figure 5:**
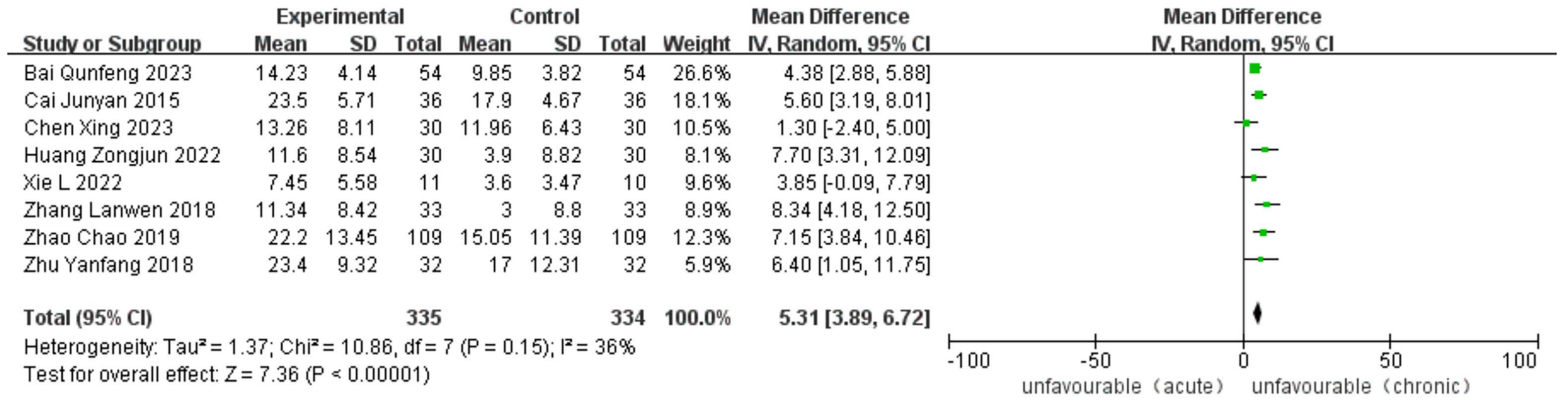
Forest plot for comparison of length of FMA between ASETG and CSETG.

**Figure 6:**
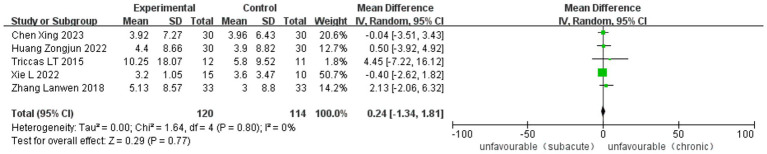
Forest plot for comparison of length of FMA between SSETG and CSETG.

Twelve studies, five RCT and seven observational studies, reported improvement in the Fugl-Meyer Assessment. The analysis included 1,283 cases (488 in ASETG, 450 in SSETG and 345 in CSETG). Pooled analysis of the two studies revealed moderate heterogeneity for the ASETG vs. SSETG comparison (*I*^2^ = 32%, *p* = 0.18) and the ASETG vs. CSETG comparison (*I*^2^ = 36%, *p* = 0.15). In contrast, the comparison between SSETG and CSETG showed no evidence of heterogeneity (*I*^2^ = 0%, *p* = 0.80). The random-effects model analysis demonstrated significantly greater improvement in FMA scores in the ASETG compared to the SSETG [WMD = 7.95, 95% CI (6.73, 9.16), *p* < 0.0001; Figure 4]. Similarly, the ASETG showed significantly better outcomes than the CSETG [WMD = 5.31, 95% CI (3.89, 6.72), *p* < 0.00001; Figure 5]. However, no significant difference was observed between the SSETG and CSETG groups [WMD = 0.24, 95% CI (−1.34, 1.81), *p* = 0.77; Figure 6].

#### 3.2.2 Upper extremity Fugl-Meyer Assessment improvement after treatment

**Figure 7:**
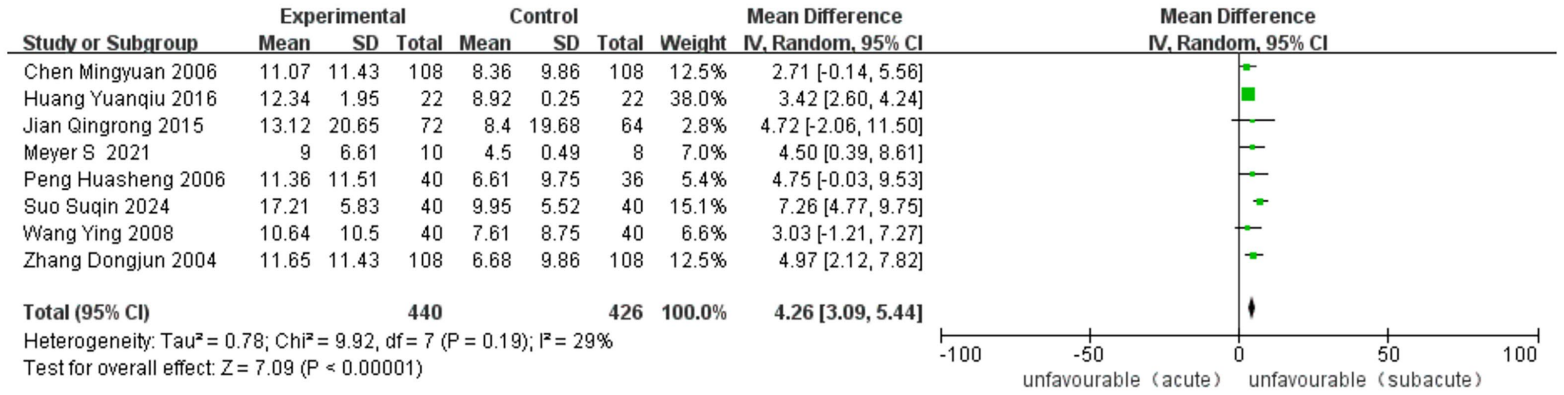
Forest plot for comparison of length of FMA-UE between ASETG and SSETG.

Thirteen studies, five RCT and eight observational studies, reported improvement in the FMA-UE. The analysis included 2,984 cases (1,319 in ASETG, 1058 in SSETG and 607 in CSETG). Low heterogeneity was observed (I^2^ = 29%, *p* = 0.19). The ASETG showed significantly better outcomes than the SSETG [WMD = 4.26, 95% CI (3.09, 5.44), *p* < 0.00001; Figure 7].

#### 3.2.3 Lower extremity Fugl-Meyer Assessment improvement after treatment

**Figure 8:**
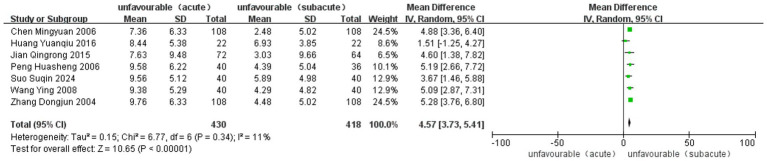
Forest plot for comparison of length of FMA-LE between ASETG and SSETG.

Nine studies, one RCT and eight observational studies, reported improvements in the FMA-LE after treatment. The analysis included 1,068 cases (480 in ASETG, 468 in SSETG and 120 in CSETG). Low heterogeneity was observed (*I*^2^ = 11%, *p* = 0.34). The ASETG showed significantly better outcomes than the SSETG [WMD = 4.57, 95% CI (3.73, 5.41), *p* < 0.00001; Figure 8].

#### 3.2.4 Berg Balance Scale improvement after treatment

**Figure 9:**
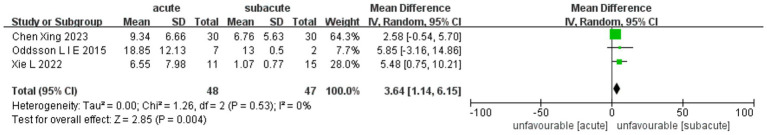
Forest plot for comparison of BBS improvement after treatment between ASETG and SSETG.

Four studies reported improvements in the BBS, including one RCTs and three observational study, involving 218 cases (48 in ASETG, 90 in SSETG and 80 in CSETG). No evidence of heterogeneity was found (*I*^2^ = 0%, *p* = 0.53). The random-effect model analysis indicated that the improvement in the BBS was significantly higher in ASETG compared to SSETG [WMD = 3.64, 95% CI (1.14, 6.15), *p* = 0.004; Figure 9].

#### 3.2.5 Modified Barthel Index improvement after treatment

**Figure 10:**
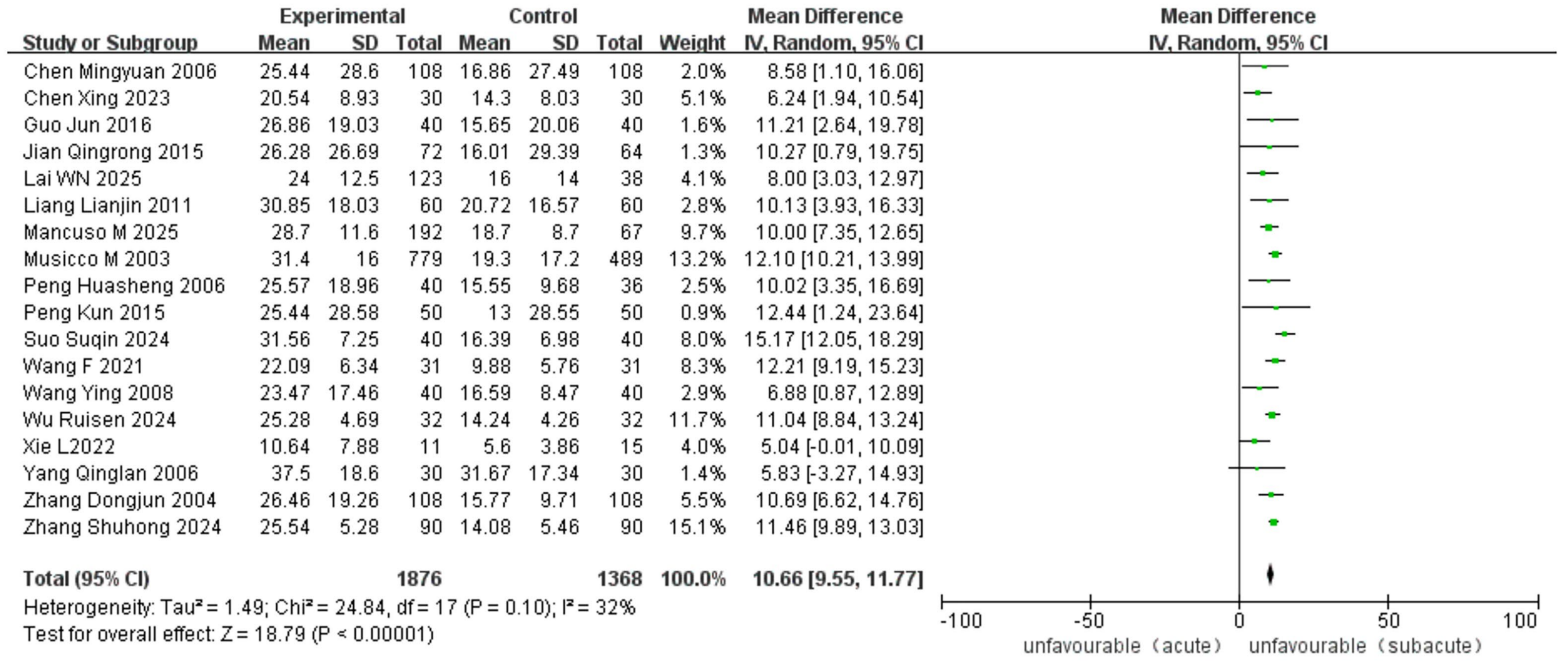
Forest plot for comparison of MBI between ASETG and SSETG.

**Figure 11:**
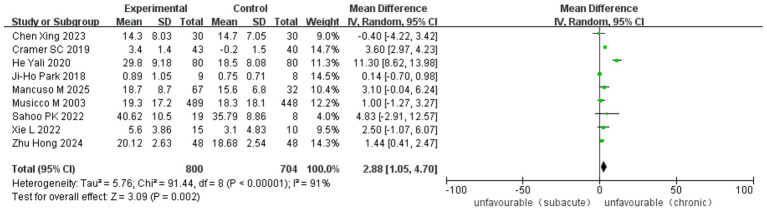
Forest plot for comparison of MBI between SSETG and CSETG.

Ten RCT and 18 observational studies reported improvements in the MBI. The analysis included 3,065 cases (1,416 in ASETG, 1050 in SSETG and 599 in CSETG). Pooled analysis revealed moderate heterogeneity for the ASETG versus SSETG comparison (I^2^ = 32%, *p* = 0.10), whereas high heterogeneity was detected for the SSETG versus CSETG comparison (*I*^2^ = 91%, *p* < 0.00001). According to the random-effects model analysis, the improvement in MBI was significantly greater for the ASETG versus SSETG [WMD = 10.66, 95% CI (9.55, 11.77), *p* < 0.00001; Figure 10] and for the SSETG versus CSETG [WMD = 2.88, 95% CI (1.05, 4.70), *p* = 0.002; Figure 11].

#### 3.2.6 Modified Ashworth Scale improvement after treatment

**Figure 12:**
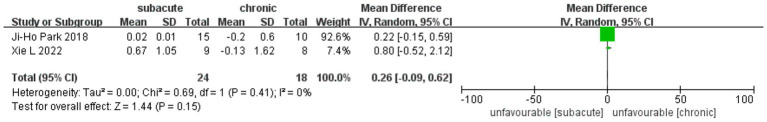
Forest plot for comparison of MAS between SSETG and CSETG.

One RCT and one observational study reported improvements in the MAS. The analysis included 42 cases (24 in SSETG and 18 in CSETG). No evidence of heterogeneity was found (*I*^2^ = 0%, *p* = 0.41). The random-effect model analysis showed no significant difference in MAS improvement between the two groups [WMD = 0.26, 95% CI (−0.09, 0.62), *p* = 0.15; Figure 12].

#### 3.2.7 Action Research Arm Test improvement after treatment

**Figure 13:**
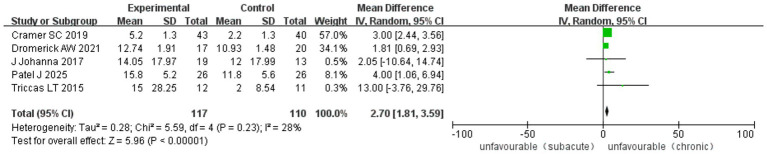
Forest plot for comparison of ARAT improvement after treatment between SSETG and CSETG.

A total of six RCTs reported improvements in the ARAT after treatment. The analysis included 1894 cases: 805 patients in SSETG, 571 in SSETG and 518 in CSETG. Pooled analysis of the six studies comparing SSETG and CSETG showed low heterogeneity (*I*^2^ = 28%, *p* = 0.43). The random-effect model analysis showed a significantly higher improvement in the ARAT in SSETG compared to CSETG [WMD = 2.70, 95% CI (1.81, 3.59), *p* < 0.00001; Figure 13].

#### 3.2.8 Subgroup analysis by intervention type

**Figure 14:**
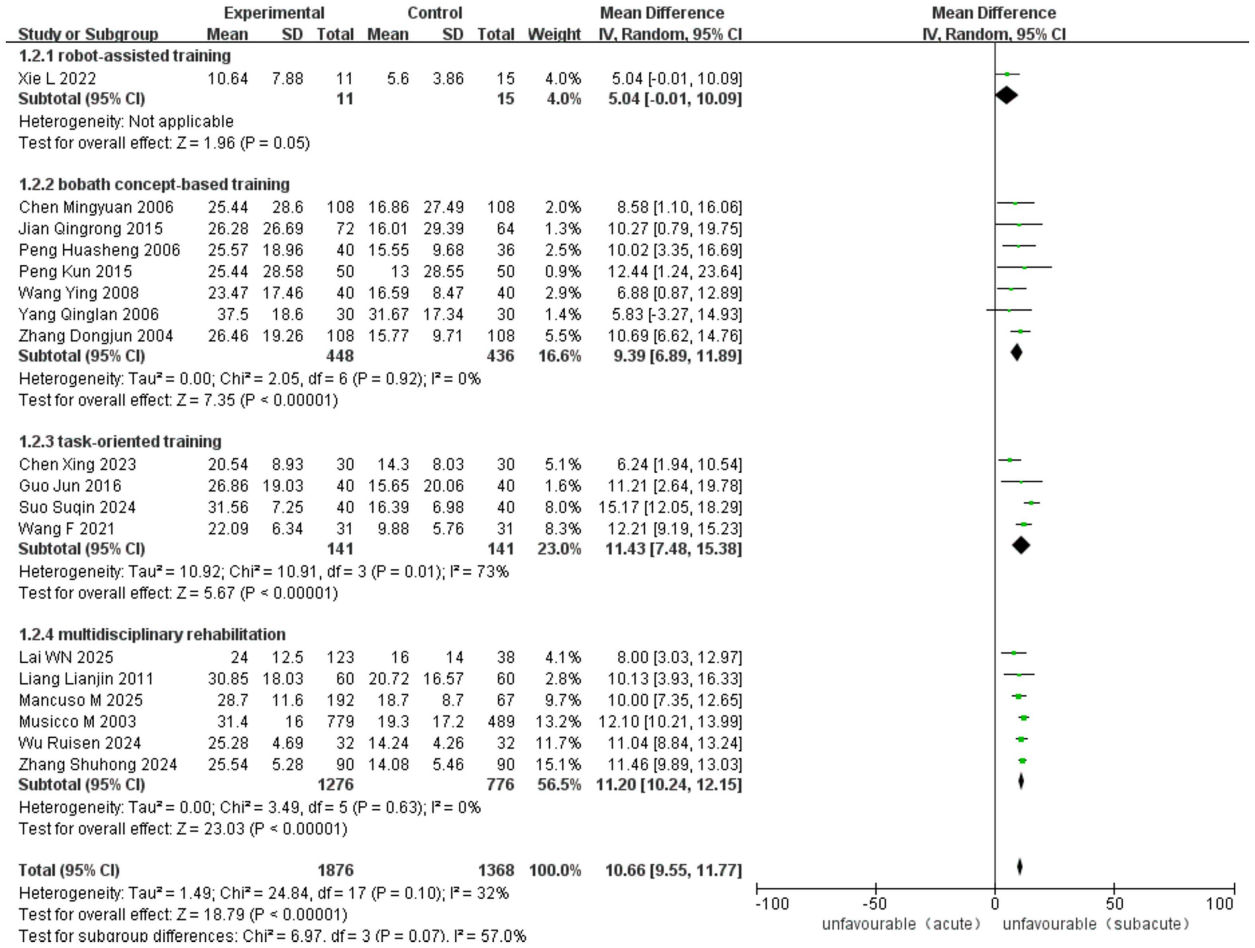
Forest plot for subgroup analysis of MBI by intervention type.

The overall pooled analysis demonstrated a significant benefit of early rehabilitation across all studies [MD = 10.66, 95% CI (9.55–11.77), *p* < 0.00001], with moderate heterogeneity (*I*^2^ = 32%, *p* = 0.10). When stratified by intervention type, no heterogeneity was observed within the bobath concept-based training subgroup (*I*^2^ = 0%, *p* = 0.92) or the multidisciplinary rehabilitation subgroup (*I*^2^ = 0%, *p* = 0.63). In contrast, substantial heterogeneity was present among studies employing task-oriented training (*I*^2^ = 73%, *p* = 0.01). The robot-assisted training subgroup included only one study, precluding assessment of heterogeneity. All subgroups favored early rehabilitation, with no significant difference between subgroups (*p* = 0.07) (Figure 14).

#### 3.2.9 Subgroup analysis by stroke type

**Figure 15:**
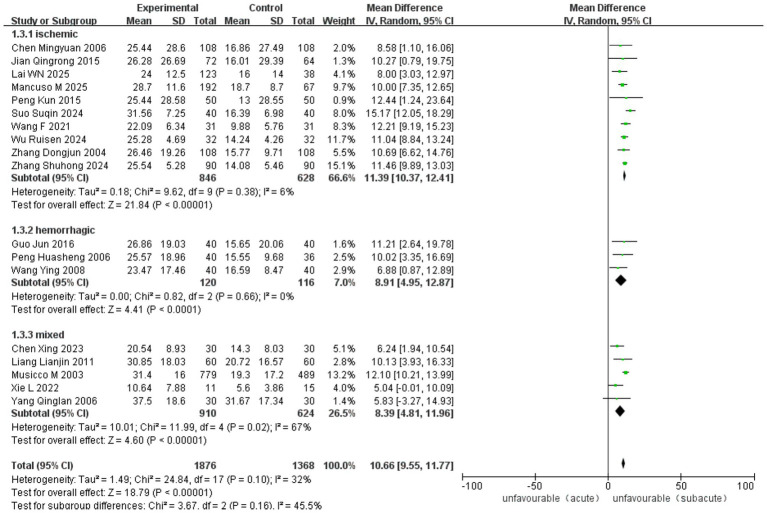
Forest plot for subgroup analysis of MBI by stroke type.

The overall pooled analysis demonstrated a significant benefit of early rehabilitation across all studies (MD = 10.66, 95% CI [9.55–11.77], *p* < 0.00001), with moderate heterogeneity (*I*^2^ = 32%, *p* = 0.10). When stratified by stroke type, no heterogeneity was observed within the ischemic subgroup (*I*^2^ = 6%, *p* = 0.38) or the hemorrhagic subgroup (*I*^2^ = 0%, *p* = 0.66). In contrast, moderate heterogeneity was present among studies in the mixed stroke subgroup (*I*^2^ = 67%, *p* = 0.02). All subgroups favored early rehabilitation, with no significant difference between subgroups (*p* = 0.16) (Figure 15).

#### 3.2.10 Subgroup analysis by total treatment dose

**Figure 16:**
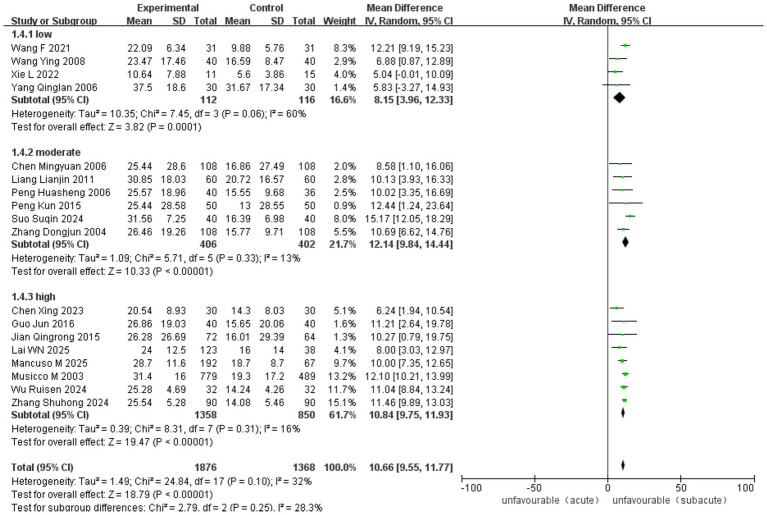
Forest plot for subgroup analysis of MBI by total treatment dose.

The overall pooled analysis demonstrated a significant benefit of early rehabilitation across all studies [MD = 10.66, 95% CI (9.55–11.77), *p* < 0.00001], with moderate heterogeneity (*I*^2^ = 32%, *p* = 0.10). When stratified by total treatment dose, low heterogeneity was observed within the moderate-dose subgroup (*I*^2^ = 13%, *p* = 0.33) and the high-dose subgroup (*I*^2^ = 16%, *p* = 0.31). In contrast, substantial heterogeneity was present among studies in the low-dose subgroup (*I*^2^ = 60%, *p* = 0.06). All subgroups favored early rehabilitation, with no significant difference between subgroups (*p* = 0.25) (Figure 16).

### 3.3 GRADE evidence quality assessment

As summarized in Table 1, in the comparisons of acute versus subacute phase and acute versus chronic phase, most core outcomes (e.g., FMA, MBI) showed clear effect sizes and were rated as moderate certainty of evidence, with downgrading primarily due to risk of bias (e.g., inadequate randomization and lack of blinding). In contrast, for the subacute versus chronic phase comparison, the certainty of evidence for MBI was downgraded to very low due to very serious inconsistency (*I*^2^ > 75%), indicating that findings from this comparison should be interpreted with caution. Furthermore, the evidence base for outcomes such as BBS (based on only three non-randomized studies) and MAS (extremely small sample size) was relatively weak, with certainty rated as low or very low. Overall, exercise training initiated in the acute phase provides more robust benefits for functional recovery compared with the subacute or chronic phase, with higher certainty of evidence.

### 3.4 Heterogeneity and sensitivity analysis

In the comparison between acute and subacute stroke groups based on the Modified Barthel Index (MBI), subgroup analyses were conducted to investigate potential sources of heterogeneity. Leave-one-out sensitivity analysis revealed that excluding Chen et al. (38) reduced the heterogeneity in the task-oriented training subgroup from 73 to 4%, with the pooled effect size remaining stable (MD = 13.49, 95% CI [11.32 to 15.66]). The test for subgroup differences became statistically significant (from *p* = 0.07 to *p* = 0.007), indicating that this study masked the differences between intervention types. Excluding Musicco et al. (22) decreased heterogeneity in the mixed stroke subgroup from 67 to 0%, while the pooled effect size remained stable (MD = 7.87, 95% CI [4.98–10.76]). The test for subgroup differences became statistically significant (from *p* = 0.16 to *p* = 0.004), identifying this study as the primary source of heterogeneity in the mixed stroke subgroup. Excluding Wang et al. (19) reduced heterogeneity in the low-dose subgroup from 60 to 0%, with the pooled effect size changing from 8.15 [95% CI (3.96–12.33)] to 5.81 [95% CI (2.25–9.36)]. The test for subgroup differences remained statistically significant (from *p* = 0.16 to *p* = 0.01), confirming this study as a contributing source of heterogeneity in the low-dose subgroup. The direction of the overall effect remained unchanged across all analyses, demonstrating the robustness of the findings in the comparison between acute and subacute stroke groups based on MBI.

### 3.5 Publication bias

**Figure 17:**
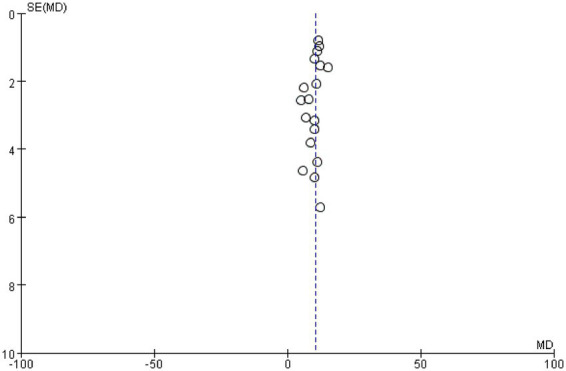
Funnel plots for publication bias assessment in MBI.

**Figure 18:**
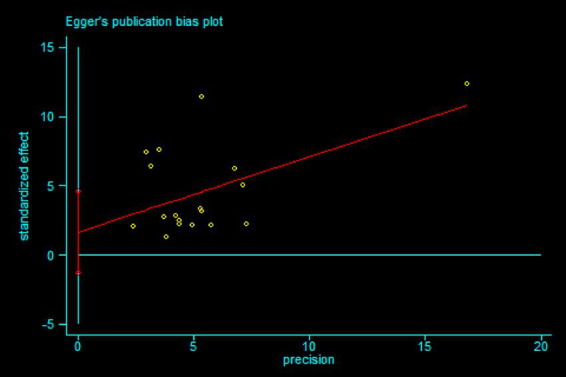
Egger’s publication bias plot in MBI.

We constructed the funnel plot and Egger’s test for MBI to explore the level of publication bias. Analysis of the funnel plot showed that the scatter plot was symmetrical indicating that the level of publication bias was low (Figure 17). In addition, the Egger’s test revealed that there was no evidence of publication bias in MBI data among studies (*p* = 0.265) (Figure 18).

## 4 Discussion

With the aging population increasing, the number of elderly stroke patients continues to rise (57), and younger people are also increasingly affected by stroke (58, 59). This trend contributes to a reduction in workforce productivity, and the resulting disabilities and limitations in social participation place a heavy burden on families and society. Each year, significant financial resources are allocated to treat stroke-related dysfunction (59). For patients with limb dysfunction, various rehabilitation treatments such as physical therapy, occupational therapy, exercise training, and acupuncture can be used (12). Among these, exercise training stands out as a healthy, long-term, and accessible rehabilitation method with considerable potential for application and development. Numerous studies have demonstrated that exercise training positively impacts the recovery of sensorimotor function, cognition, balance, gait, and activities of daily living in acute (3–5), subacute (6–8), and chronic (9, 10) stroke patients. However, few studies have compared the benefits of exercise training across these different stages. Some research suggests that earlier rehabilitation is not always better, as premature rehabilitation can increase oxygen consumption, activate the inflammatory cascade, promote apoptosis, inhibit dendrite formation, impair nerve remodeling, and potentially worsen stroke outcomes (51). On the other hand, some studies argue that early rehabilitation and ultra-early rehabilitation (60) can more effectively improve dysfunction, accelerate recovery of limb function, reduce complications from prolonged bed rest, prevent muscle atrophy, spasticity, and contractures, improve joint mobility, and enhance self-care abilities. This study aimed to determine the optimal timing for stroke rehabilitation intervention and provide evidence-based guidance for clinical decision-making in early stroke rehabilitation.

Studies have shown that the recovery of lower limb function in stroke patients is generally better than that of upper limb (61, 62). This is because the motor and sensory cortex of the lower limbs and some of their conductive fibers are located near the midline of the brain, where blood supply comes from both the anterior and middle cerebral arteries. As a result, the brain tissue responsible for lower limb movement and sensation receives more blood supply, increasing its chance of survival (62). This leads to easier restoration of lower limb function. Therefore, upper limb recovery is often the most challenging aspect of rehabilitation, and its improvement is frequently used as a key indicator of treatment effectiveness. The Action Research Arm Test (ARAT) is an internationally accepted assessment tool for upper limb function, measuring the ability to handle objects of varying sizes, weights, and shapes (63). The pooled analysis of five studies demonstrated that exercise training initiated in the acute stage significantly improved upper limb function compared to that initiated in the chronic stage [MD = 2.70, 95% CI (1.81–3.59), *p* < 0.00001], with low heterogeneity (*I*^2^ = 28%, *p* = 0.23). This finding suggests that early intervention confers greater benefits for upper limb recovery, supporting the recommendation to initiate exercise training as early as possible after stroke. Choi et al. (64) also found that task-oriented training significantly improved upper limb function, visual perception, and self-care ability in acute stroke patients after 6 weeks of exercise training. Other studies (65) have shown that acute stroke patients with anxiety and depression not only improved their limb function after 12 weeks of exercise training but also experienced improvements in mental and emotional health.

Balance function refers to the body’s ability to maintain a stable sitting or standing position, which involves resisting gravity to maintain posture (66). Balance relies on sensory input, central integration, and motor regulation. Sensory input includes proprioceptive, visual, and vestibular feedback, while motor regulation involves ankle, hip, and stepping strategies (66). Because balance function involves multiple systems, restoring balance is a key measure of the effectiveness of exercise training. The Berg Balance Scale (BBS) is the most widely used assessment tool for balance in stroke patients and has shown good reliability, validity, and sensitivity across different recovery stages (67). BBS consists of 14 tasks, ranging from easy to difficult, with each task scored from 0 to 4, for a total score of 56 points. Higher scores indicate better balance. A score ≤50 suggests a risk of falling, while a score ≤40 indicates nearly a 100% fall risk (68). Kim et al. (69) found that combining robot-assisted gait training with conventional physical therapy during the acute phase of cerebral infarction significantly improved balance dysfunction, with the experimental group showing greater improvement in BBS scores compared to those receiving only conventional therapy. Cabanas-Valdes et al. (70) conducted a randomized controlled trial on 79 subacute stroke patients and found that core stability training combined with standard physical therapy significantly improved sitting and standing balance as well as gait. After 5 weeks, the experimental group’s BBS scores increased by an average of 14.54 points compared to the control group. Lund et al. (71) found that resistance and aerobic training during the chronic phase of cerebral infarction improved patients’ balance, gait speed, and six-minute walking distance. These findings suggest that rehabilitation is a long-term process, with significant benefits even during the chronic phase, emphasizing the need for lifelong rehabilitation. However, these studies did not compare the effectiveness of exercise rehabilitation at different stages of stroke. The pooled analysis suggested that patients in the acute stroke group may achieve greater improvement in balance function, as measured by the Berg Balance Scale (BBS), compared to those in the subacute group [MD = 4.25, 95% CI (1.39–7.11), *p* = 0.004], with moderate heterogeneity (*I*^2^ = 48%, *p* = 0.15). Balance recovery after stroke involves complex interactions between sensory, motor, and cognitive systems. However, this finding should be interpreted with caution due to the limited number of studies included in the analysis, which may have resulted in insufficient statistical power and compromised the robustness of the results. The optimal intensity, frequency, and duration of balance-oriented exercise programs remain to be established, and future research with larger sample sizes and standardized protocols is needed to confirm these preliminary findings.

The Fugl-Meyer Assessment (FMA) is a motor function assessment tool specifically designed for stroke patients. It was further developed by Fugl-Meyer et al., based on the six-level functional classification of the Brunnstrom scale (72). The FMA includes assessments for both upper and lower limb movements. The upper limb section consists of 33 items, and the lower limb section consists of 17 items, with each item scored out of 2 points. The total score is 100, where scores below 50 indicate severe motor impairment. A score of 50–84 indicates significant impairment, 85–95 indicates moderate impairment, and 96–99 indicates mild impairment (73). The FMA provides a clear understanding of a patient’s motor function, helping clinicians design and adjust rehabilitation plans to achieve the best possible outcomes. Wu et al. (74) conducted a 12-week randomized controlled trial on 61 acute stroke patients and found that a collaborative nursing model based on tele-rehabilitation significantly improved FMA scores, while patients receiving conventional rehabilitation and nursing guidance showed less improvement. Kelley et al. (75) emphasized the importance of early intervention in exercise training to prevent bed rest complications. Muscle mass begins to decrease within 24 h of inactivity, with the most rapid decline occurring after 5–7 days. Blood volume decreases by 5% after 1 day and by 20% after 14 days, eventually leading to muscle atrophy, tendon contracture, and bone loss. Zhang et al. (6) conducted a randomized controlled trial on 34 subacute stroke patients and found that robot-assisted gait training significantly improved FMA scores, with lower limb improvements being more pronounced than those in the upper limbs. However, long-term exercise training was less effective in improving FMA levels in chronic stroke patients (76), as functional recovery gradually declines (77). Our meta-analysis indicated that the ASETG showed significantly greater improvements in FMA scores compared to the SSETG. This trend was also observed in the FMA-UE and FMA-LE subscales when compared to the SSETG. Furthermore, the ASETG outperformed the CSETG in FMA scores. Our findings confirm the importance of early rehabilitation and we hope that more research will confirm this fact in the future.

Muscle spasm after stroke is caused by an increased stretch reflex and heightened muscle tension, commonly manifesting as increased muscle tone in the hemiplegic limbs (78). While spasticity in the lower limbs can be beneficial for standing and walking, as well as preventing deep vein thrombosis, excessive spasticity can limit joint movement, leading to stiffness and tendon contractures (78). The Modified Ashworth Scale (MAS) is used to measure the degree of muscle spasticity by assessing resistance encountered during passive joint movement (79). Rehabilitation often uses exercise training to reduce spasticity in stroke patients. Studies have found that combining extracorporeal shock wave therapy with isokinetic strength training significantly reduces limb spasticity. One study of 45 acute stroke patients undergoing 4 weeks of exercise training found that MAS scores significantly decreased in the experimental group, while shock wave therapy alone had less effect (80). Opheim et al. (81) followed 117 stroke patients for 1 year and found that 25% of patients experienced limb spasticity by the third day, and 46% by the twelfth day, leading to worsened sensorimotor function, increased pain, and reduced joint range of motion. Liu et al. (82) found that motion training based on the Maitland mobilization technique significantly improved spasticity in stroke patients, accelerated progress in Brunnstrom staging, and promoted joint and isolated movements. Park et al. (83) conducted a randomized controlled trial on 34 chronic stroke patients and found that transcutaneous electrical nerve stimulation combined with exercise training significantly improved MAS scores, balance, and gait, while preventing complications such as foot drop and tendon contracture. While exercise training can effectively reduce spasticity in acute, subacute, and chronic stroke patients, it remains unclear which stage of training is most effective. The pooled analysis found no significant difference in spasticity reduction, as measured by the Modified Ashworth Scale (MAS), between patients who received exercise training in the subacute stage and those who received it in the chronic stage. However, this finding should be interpreted with caution due to the limited number of studies included in this analysis. Although a total of 38 studies were included in this systematic review, the number of studies available for each individual meta-analysis was substantially smaller—only two studies were eligible for this comparison—which may have resulted in insufficient statistical power and compromised the robustness of the results. These limitations should be acknowledge preliminary findings and to better understand the effects of exercise training timing on spasticity after stroke.edged when drawing clinical implications. Future research with larger sample sizes is needed to confirm these.

The MBI is an assessment tool for daily living abilities developed by Shah et al. (84). It addresses limitations in the original Barthel Index, such as low sensitivity and rough classifications. MBI retains the content of the Barthel Index but weights the ratings and subdivides 10 items into five levels: complete dependence, maximum help, moderate help, minimum help, and complete independence (84). Through MBI assessment, the basic status of a patient’s daily living ability in various settings, such as the home, healthcare facilities, and community, can be clearly understood. MBI can also assess the effectiveness of rehabilitation treatment and provide a basis for setting goals and designing treatment plans. Studies have shown that task-oriented exercise training in the acute phase of stroke significantly improves MBI and aids patients in returning to their families and society (84). Another study found that rehabilitation robot-based exercise training significantly improved MBI in subacute stroke patients. In this study, 35 patients received 4 weeks of exercise training, and the experimental group showed significant improvement in MBI, while patients using conventional rehabilitation equipment showed less progress (85). Park et al. (86) conducted a randomized controlled trial on 30 chronic stroke patients and found that trunk-based exercise training, both land-based and aquatic, improved MBI. The study also suggested that applying robotic rehabilitation training to subacute stroke patients could lead to more effective outcomes in the future. However, previous studies have not extensively compared the effectiveness of exercise training across different stroke stages. Our meta-analysis found that the ASETG exhibited significantly greater improvements in MBI scores compared to the SSETG. However, there was no significant difference in MBI improvement between SSETG and CSETG. Our research highlights the importance of acute rehabilitation therapy, but over time, improvements in MBI during the subacute and chronic phases gradually reach a plateau. Additionally, there are limited studies comparing exercise training across the acute, subacute, and chronic stages, leading to significant publication bias. In addition, the safety of exercise training in the acute phase is also debated, with many neurologists and neurosurgeons hesitant to prescribe early rehabilitation, resulting in many patients not receiving timely treatment during the acute phase.

## 5 Limitation

This study had several limitations. First, only 16 RCTs and 22 observational study were included, leading to inevitable bias. Second, there are few studies that compare the effects of exercise training across all three stroke phases—acute, subacute, and chronic. Many studies only examine two of these phases. Future meta-analyses should aim for larger sample sizes, better study designs, and multi-center RCTs. Third, although 38 studies were included in this systematic review, the number of studies available for each meta-analysis was substantially smaller. Specifically, the analyses for MAS, ARAT, and BBS were each based on a limited number of studies, which may have resulted in insufficient statistical power and compromised the robustness of the findings. Therefore, these results should be interpreted with caution. Future large-scale studies are needed to validate these preliminary findings. Fourth, although subgroup analyses were conducted for exercise type, intervention dose, and stroke type, other important variables—such as stroke severity and type of control group intervention—could not be examined due to insufficient data in the included studies. Finally, pooled results should be interpreted cautiously due to variations in patient profiles, case numbers, and ethnic backgrounds across studies.

## 6 Conclusion

In conclusion, this meta-analysis provides supportive evidence that early initiation of exercise training after stroke is associated with improved functional recovery, as measured by FMA, BBS, MBI, and ARAT. While these findings reinforce the potential benefits of early rehabilitation, they must be interpreted within the context of the inherent heterogeneity and limited number of studies included. The lack of significant effect on spasticity (MAS) warrants further investigation. To strengthen the evidence base and establish definitive clinical guidelines, future research should prioritize large-scale, methodologically robust randomized controlled trials with long-term follow-up.

## Data Availability

The original contributions presented in the study are included in the article/Supplementary material, further inquiries can be directed to the corresponding author.

## References

[1] Martin SS Aday AW Almarzooq ZI Anderson CAM Arora P Avery CL. 2024 heart disease and stroke statistics: a report of US and global data from the American Heart Association. Circulation. (2024) 149:e561–7. doi: 10.1161/cir.0000000000001209, 38264914 PMC12146881

[2] Stinear CM Lang CE Zeiler S Byblow WD. Advances and challenges in stroke rehabilitation. Te Lancet Neurol. (2020) 19:348–60. doi: 10.1016/s1474-4422(19)30415-6, 32004440

[3] Abe T Iwata K Yoshimura Y Shinoda T Inagaki Y Ohya S. Low muscle mass is associated with walking function in patients with acute ischemic stroke. J Stroke Cerebrovasc Dis. (2020) 29:105259–66. doi: 10.1016/j.jstrokecerebrovasdis.2020.105259, 33066891

[4] Strommen AM Christensen T Jensen K. Intensive treadmill training in the acute phase after ischemic stroke. Int J Rehabil Res. (2016) 2016:145–52. doi: 10.1097/mrr.0000000000000158 26926203

[5] Buvarp D Viktorisson A Axelsson F Lehto E Lindgren L. Physical activity trajectories and functional recovery after acute stroke among adults in Sweden. JAMA Netw Open. (2023) 2023:348–60. doi: 10.1001/jamanetworkopen.2023.10919, 37126346 PMC10152305

[6] Zhang H Li X Gong Y Wu J Chen J Chen W. Three-dimensional gait analysis and sEMG measures for robotic-assisted gait training in subacute stroke: a randomized controlled trial. Biomed Res Int. (2023) 2023:1–12. doi: 10.1155/2023/7563802, 37082189 PMC10113045

[7] Hogg S Holzgraefe M Wingendorf I Mehrholz J Herrmann C Obermann M. Upper limb strength training in subacute stroke patients: study protocol of a randomised controlled trial. Trials. (2019) 2019:168–79. doi: 10.1186/s13063-019-3261-3, 30876438 PMC6420769

[8] Ranzani R Lambercy O Metzger J-C Califfi A Regazzi S Dinacci D. Neurocognitive robot-assisted rehabilitation of hand function: a randomized control trial on motor recovery in subacute stroke. J Neuroeng Rehabil. (2020) 2020:115–28. doi: 10.1186/s12984-020-00746-7, 32831097 PMC7444058

[9] Macko R. Task-oriented aerobic exercise in chronic hemiparetic stroke:training protocols and treatment effects. Top Stroke Rehabil. (2005) 12:45–57. doi: 10.1310/PJQN-KAN9-TTVY-HYQH, 15736000

[10] Chang K-W Lin C-M Yen C-W Yang C-C Tanaka T Guo L-Y. The effect of walking backward on a treadmill on balance, speed of walking and cardiopulmonary fitness for patients with chronic stroke: a pilot study. Int J Environ Res Public Health. (2021) 18:2376–86. doi: 10.3390/ijerph18052376, 33804374 PMC7967772

[11] Society of Neurolog C, Society of Neurorehabilitation C, Society of Cerebrovascular Disease C. Chinese guidelines for early rehabilitation after stroke. Chin J Neurol. (2017) 50:405–12. doi: 10.3760/cma.j.issn.1006-7876.2017.06.002

[12] Winstein CJ Stein J Arena R Bates B Cherney LR Cramer SC. Guidelines for adult stroke rehabilitation and recovery. Stroke. (2016) 47:e98–e169. doi: 10.1161/str.0000000000000098, 27145936

[13] Park J-H Shin Y-I You JH Park MS. Comparative effects of robotic-assisted gait training combined with conventional physical therapy on paretic hip joint stiffness and kinematics between subacute and chronic hemiparetic stroke. NeuroRehabilitation. (2018) 42:181–90. doi: 10.3233/nre-172234, 29562554

[14] Martinuzzi A Jonsdottir J Thorsen R Aprile I Galeri S Spannocchi G. Arm rehabilitation in post stroke subjects: a randomized controlled trial on the efficacy of myoelectrically driven FES applied in a task-oriented approach. PLoS One. (2017) 12:e0188642–58. doi: 10.1371/journal.pone.0188642, 29200424 PMC5714329

[15] Meyer S Verheyden G Kempeneers K Michielsen M. Arm-hand boost therapy during inpatient stroke rehabilitation: a pilot randomized controlled trial. Front Neurol. (2021) 12:652042–53. doi: 10.3389/fneur.2021.652042, 33716948 PMC7952763

[16] Dromerick AW Geed S Barth J Brady K Giannetti ML Mitchell A. Critical period after stroke study (CPASS): a phase II clinical trial testing an optimal time for motor recovery after stroke in humans. Proc Natl Acad Sci USA. (2021) 118:1–10. doi: 10.1073/pnas.2026676118, 34544853 PMC8488696

[17] Triccas LT Burridge JH Hughes A Verheyden G Desikan M Rothwell J. A double-blinded randomised controlled trial exploring the effect of anodal transcranial direct current stimulation and uni-lateral robot therapy for the impaired upper limb in sub-acute and chronic stroke. NeuroRehabilitation. (2015) 37:181–91. doi: 10.3233/nre-151251, 26484510

[18] Cramer SC Dodakian L Le V See J Augsburger R McKenzie A. Efficacy of home-based telerehabilitation vs in-clinic therapy for adults after stroke. JAMA Neurol. (2019) 76:1079–86. doi: 10.1001/jamaneurol.2019.1604, 31233135 PMC6593624

[19] Wang F Zhang S Zhou F Zhao M Zhao H. Early physical rehabilitation therapy between 24 and 48 h following acute ischemic stroke onset: a randomized controlled trial. Disabil Rehabil. (2021) 44:3967–72. doi: 10.1080/09638288.2021.1897168, 33736542

[20] Patel J Qiu Q Fluet GG Gorin H Gutterman J Karunakaran K. A randomized controlled trial of timing and dosage of upper extremity rehabilitation in virtual environments in persons with subacute stroke. Sci Rep. (2025) 15:13834–49. doi: 10.1038/s41598-025-98618-4, 40263476 PMC12015485

[21] Mancuso M Iosa M Morone G De Bartolo D Ciancarelli I. How do the timing of early rehabilitation together with cognitive and functional variables influence stroke recovery? Results from the CogniReMo Italian multicentric study. Healthcare. (2025) 13:316–27. doi: 10.3390/healthcare13030316, 39942505 PMC11817751

[22] Musicco M Emberti L Nappi G Caltagirone C. Early and long-term outcome of rehabilitation in stroke patients: the role of patient characteristics, time of initiation, and duration of interventions. Arch Phys Med Rehabil. (2003) 84:551–8. doi: 10.1053/apmr.2003.50084 12690594

[23] Sahoo PK Nehal N. Effect of late-onset stroke rehabilitation on medical morbidities and functional recovery: a single-center observational study. Cureus. (2022) 14:e33002–14. doi: 10.7759/cureus.33002, 36712731 PMC9879282

[24] Lai WN Tan CYF Ong CY Wong MSJ Low LL. Predictors of short-term functional recovery in ischemic stroke rehabilitation at community hospitals in Singapore. Front Stroke. (2025) 4:1704636–48. doi: 10.3389/fstro.2025.1704636, 41541862 PMC12802713

[25] Suqin S Yan G Xuemei T. Research on the application of rehabilitation exercise timing in acute ischemic stroke. J Bengbu Med Coll. (2024) 49:1249–55. doi: 10.13898/j.cnki.issn.1000-2200.2024.09.028

[26] Ruisen W Shujie L. The effects of rehabilitation treatment at different times on the neurological function and motor ability of patients with cerebral infarction and hemiplegia. J Med Inform. (2024) 37:139–42. doi: 10.3969/j.issn.1006-1959.2024.06.024

[27] Shuhong Z Yingchun L Gaoyang S. Comparison of the effects of different timing rehabilitation treatments on neurological function in elderly patients with cerebral infarction. Chin Gen Pract Med. (2022) 20:678–95. doi: 10.16766/j.cnki.issn.1674-4152.002428

[28] Qunfeng B Yongfang L Lin Q Jie D. The impact of rehabilitation treatment at different times on the neurological function and motor ability of patients with cerebral infarction and hemiplegia. Clin Med Eng. (2023) 30:39–40. doi: 10.3969/j.issn.1674-4659.2023.01.0039

[29] Oddsson LIE Finkelstein MJ Meissner S. Feasibility of early functional rehabilitation in acute stroke survivors using the balance-bed-a technology that emulates microgravity. Front Syst Neurosci. (2015) 9:83–92. doi: 10.3389/fnsys.2015.00083, 26074789 PMC4445307

[30] Xie L Yoon BH Park C You JH. Optimal intervention timing for robotic-assisted gait training in hemiplegic stroke. Brain Sci. (2022) 12:1058–70. doi: 10.3390/brainsci12081058, 36009121 PMC9405763

[31] Mingyuan C Cuihong M. Study on the influence of early and late rehabilitation treatment on functional recovery of cerebral infarction patients. Chin Nurs Res. (2006) 20:2939–40. doi: 10.3969/j.issn.1004-7115.2009.11.009

[32] Feng T Qiang H Bei Z Yulong B Yi W Yongshan W. Effects of rehabilitation on hand function and activities of daily living in patients with cerebral hemorrhage at different time of intervention. Chin J Rehabil Theory Pract. (2012) 18:501–4. doi: 10.3969/j.issn.1006-9771.2012.06.001

[33] Jun G Fanglian M. The effects of different rehabilitation interventions on neurological function and daily living ability of patients with cerebral hemorrhage were investigated. Nurs Rehabil. (2016) 1:163–3. doi: 10.19347/j.cnki.2096-1413.2016.19.091

[34] Kun P Pengcheng Z. Effect of different rehabilitation training intervention time on limb function recovery of cerebral infarction patients2015. Chin Med Guide. (2015) 13:95–6. doi: 10.15912/j.cnki.gocm.2015.02.068

[35] Yali H Xiangcheng Z Hon L. Study on the correlation between different intervention time of rehabilitation treatment and rehabilitation effect of hemiplegia patients after cerebral infarction. Chin J Pract Nerv Dis. (2020) 23:2187–92. doi: 10.12083/SYSJ.2021.01.010

[36] Huasheng P Chunlan Y. Effect of rehabilitation intervention time on functional recovery of patients with cerebral hemorrhage. Chin J Rehabil Theory Pract. (2006) 12:150–19. doi: 10.3969/j.issn.1006-9771.2006.02.027

[37] Qinglan Y Xiaoyan G Hai L Tao D. Study on the effect of rehabilitation therapy on motor function of stroke patients at different stages. Hebei Med J. (2006) 28:468–9. doi: 10.3969/j.issn.1673-6567.2010.05.015

[38] Xing C. Study on the effect of rehabilitation therapy on motor function of stroke patients at different stages. Chin Sci Technol J Database. (2022) 1:126–8.

[39] Yanfang Z. Influence of intervention time of rehabilitation therapy on rehabilitation effect of cerebral infarction patients with hemiplegia. Chin J Trauma Disab Med. (2018) 1:86–7. doi: 10.13214/j.cnki.cjotadm.2018.21.060

[40] Hong Z. Analysis of the influence of rehabilitation intervention time on rehabilitation effect of cerebral infarction patients with hemiplegia. Chin Sci Technol J Database Med. (2024) 1:5–8.

[41] Chao Z. The influence of intervention time of rehabilitation therapy on rehabilitation effect of cerebral infarction patients with hemiplegia. Clin Res. (219) 27:90–1.

[42] Xiaofei Y. Effect of rehabilitation intervention time on prognosis of cerebral infarction patients with hemiplegia. Chin Sci Technol J Database Med. (2024) 1:163–6.

[43] Lanwen Z. Evaluation of clinical correlation between interventional time and therapeutic effect of rehabilitation treatment for cerebral hemorrhage. Mod Diagn Treat. (2018) 29:2114–6.

[44] Zongjun H. Clinical correlation study of intervention time and therapeutic effect in rehabilitation treatment of cerebral hemorrhage. Chin Sci Technol J Database. (2022) 1:292–5.

[45] Yuanqiu H. Clinical correlation study of intervention time and therapeutic effect in rehabilitation treatment of cerebral hemorrhage. China Med Pharm. (2016) 6:198–201.

[46] Junyan C Guangyu S. Observation on the curative effect of early comprehensive rehabilitation for patients with cerebral stroke. Basic Clin Med. (2015) 35:1531–4.

[47] Lianjin L Weiping P Jian G. Influence of early and late rehabilitation training on ADL ability in elderly patients with stroke. Chin J Rehabil Med. (2011) 26:984–5. doi: 10.3969/j.issn.1001-1242.2011.10.025

[48] Qingrong J Xiuling W Ling L. To explore the effect of early and late rehabilitation on functional recovery of cerebral infarction patients. Chin J Pract Nerv Dis. (2015) 19:64–5.

[49] Dongjun Z Shiwen Z Guixiang C Sujun L Yizhao L. A comparative study of the effects of early and late rehabilitation on functional recovery of cerebral infarction patients. Chin J Rehabil Med. (2004) 19:588–90. doi: 10.3969/j.issn.1672-5085.2009.08.001

[50] Ying W Xuefeng W Yan L. Effect of early rehabilitation intervention on functional recovery of patients with cerebral hemorrhage. HEI Long Jiang Med J. (2008) 32:441–2. doi: 10.3969/j.issn.1004-5775.2008.06.024

[51] Li F Pendy JT Ding JN Peng C Li X Shen J. Exercise rehabilitation immediately following ischemic stroke exacerbates inflammatory injury. Neurol Res. (2017) 39:530–7. doi: 10.1080/01616412.2017.1315882, 28415917

[52] Shen J Huber M Zhao EY Peng C Li F Li X. Early rehabilitation aggravates brain damage after stroke via enhanced activation of nicotinamide adenine dinucleotide phosphate oxidase (NOX). Brain Res. (2016) 1648:266–76. doi: 10.1016/j.brainres.2016.08.001, 27495986

[53] Larissa Shamseer D Davina Ghersi M Mark Petticrew A. Preferred reporting items for systematic review and meta-analysis protocols (PRISMA-P) 2015 statement. Syst Rev. (2015) 4:1–9. doi: 10.1186/2046-4053-4-1, 25554246 PMC4320440

[54] Alt Murphy M Munoz-Novoa M Heremans C Branscheidt M Cabanas-Valdes R Engelter ST. European stroke organisation (ESO) guideline on motor rehabilitation. Eur Stroke J. (2025) 10:1160–88. doi: 10.1177/23969873251338142, 40401760 PMC12098312

[55] Cochrane Collaboration. Cochrane Handbook for Systematic Reviews of Interventions. Chichester: Wiley-Blackwell (2008).

[56] Stang A. Critical evaluation of the Newcastle-Ottawa scale for the assessment of the quality of nonrandomized studies in meta-analyses. Eur J Epidemiol. (2010) 25:603–5. doi: 10.1007/s10654-010-9491-z, 20652370

[57] Higgins JT Deeks J. Measuring inconsistency in meta-analyses. BMJ. (2003) 327:557–60. doi: 10.1136/bmj.327.7414.557, 12958120 PMC192859

[58] George Davey Smith D Christoph Minder M. Bias in meta analysis detected by a simple, graphical test. BMJ. (1997) 315:629–34. doi: 10.1136/bmj.315.7109.629, 9310563 PMC2127453

[59] Perera KS de Sa Boasquevisque D Rao-Melacini P Taylor A Cheng A Hankey GJ. Evaluating rates of recurrent ischemic stroke among young adults with embolic stroke of undetermined source. JAMA Neurol. (2022) 79:450–8. doi: 10.1001/jamaneurol.2022.0048, 35285869 PMC8922202

[60] Liu L-L Liu P-N Li X-A Li Y-N. Ultra-early electroacupuncture rehabilitation for intravenous thrombolysis-induced cerebral infarction. Eur Rev Med Pharmacol Sci. (2023) 27:10419–26. doi: 10.26355/eurrev_202311_34316 37975365

[61] Desrosiers J Malouin F Richards C Bourbonnais D Rochette A Bravo G. Comparison of changes in upper and lower extremity impairments and disabilities after stroke. Int J Rehabil Res. (2003) 26:109–16. doi: 10.1097/01.mrr.0000070760.63544.e8, 12799604

[62] Abdullahi A Wong TWL Ng SSM. Variation in the rate of recovery in motor function between the upper and lower limbs in patients with stroke: some proposed hypotheses and their implications for research and practice. Front Neurol. (2023) 14:1–8. doi: 10.3389/fneur.2023.1225924, 37602245 PMC10435271

[63] Padilla-Magana JF Pena-Pitarch E Sanchez-Suarez I Tico-Falguera N. Quantitative assessment of hand function in healthy subjects and post-stroke patients with the action research arm test. Sensors. (2022) 22:3604–18. doi: 10.3390/s22103604, 35632013 PMC9147783

[64] Choi W. The effect of task-oriented training on upper-limb function, visual perception, and activities of daily living in acute stroke patients: a pilot study. Int J Environ Res Public Health. (2022) 19:3186–97. doi: 10.3390/ijerph19063186, 35328874 PMC8954660

[65] Aidar FJ Jaco Oliveira R Gama Matos D Chilibeck PD de Souza RF Carneiro AL A randomized trial of the effects of an aquatic exercise program on depression, anxiety levels, and functional capacity of people who suffered an ischemic stroke J Sports Med Phys Fitness (2018) 58: 1171–1177. doi: 10.23736/s0022-4707.17.07284-x 28488825

[66] Haruyama K Kawakami M Otsuka T. Effect of core stability training on trunk function, standing balance, and mobility in stroke patients. Neurorehabil Neural Repair. (2016) 31:240–9. doi: 10.1177/1545968316675431, 27821673

[67] Dorn TW Schache AG Pandy MG. Muscular strategy shift in human running: dependence of running speed on hip and ankle muscle performance. J Exp Biol. (2012) 215:1944–56. doi: 10.1242/jeb.064527, 22573774

[68] Downs S. The berg balance scale. J Physiother. (2015) 61:46. doi: 10.1016/j.jphys.2014.10.002, 25476663

[69] Kim HY Shin J-H Yang SP Shin MA Lee SH. Robot-assisted gait training for balance and lower extremity function in patients with infratentorial stroke: a single-blinded randomized controlled trial. J Neuroeng Rehabil. (2019) 16:99–110. doi: 10.1186/s12984-019-0553-5, 31358017 PMC6664752

[70] Cabanas-Valdes R Bagur-Calafat C Girabent-Farres M Caballero-Gomez FM Hernandez-Valino M Urrutia Cuchi G. The effect of additional core stability exercises on improving dynamic sitting balance and trunk control for subacute stroke patients: a randomized controlled trial. Clin Rehabil. (2016) 30:1024–33. doi: 10.1177/0269215515609414, 26451007

[71] Lund C Dalgas U Gronborg TK Andersen H Severinsen K Riemenschneider M. Balance and walking performance are improved after resistance and aerobic training in persons with chronic stroke. Disabil Rehabil. (2017) 40:2408–15. doi: 10.1080/09638288.2017.1336646, 28597708

[72] Sullivan KJ Tilson JK Cen SY Rose DK Hershberg J Correa A. Fugl-Meyer assessment of sensorimotor function after stroke. Stroke. (2011) 42:427–32. doi: 10.1161/strokeaha.110.592766, 21164120

[73] Lee HH Kim DY Sohn MK Shin Y-I Oh G-J Lee Y-S. Revisiting the proportional recovery model in view of the ceiling effect of Fugl-Meyer assessment. Stroke. (2021) 52:3167–75. doi: 10.1161/strokeaha.120.032409, 34134508

[74] Wu Z Xu J Yue C Li Y Liang Y. Collaborative care model based telerehabilitation exercise training program for acute stroke patients in China: a randomized controlled trial. J Stroke Cerebrovasc Dis. (2020) 29:105328–38. doi: 10.1016/j.jstrokecerebrovasdis.2020.105328, 33002792

[75] Kelley RE Vibulsresth S Bell L Duncan RC. Evaluation of kinetic therapy in the prevention of complications of prolonged bed rest secondary to stroke. Stroke. (1987) 18:638–42. doi: 10.1161/01.str.18.3.638, 3590257

[76] Sugg K Muller S Winstein C Hathorn D Dempsey A. Does action observation training with immediate physical practice improve hemiparetic upper-limb function in chronic stroke? Neurorehabil Neural Repair. (2015) 29:807–17. doi: 10.1177/1545968314565512, 25613984

[77] Andrushko JW Rinat S Greeley B Larssen BC Jones CB Rubino C. Improved processing speed and decreased functional connectivity in individuals with chronic stroke after paired exercise and motor training. Sci Rep. (2023) 13:13652–65. doi: 10.1038/s41598-023-40605-8, 37608062 PMC10444837

[78] Persson CU Holmegaard L Redfors P Jern C Blomstrand C Jood K. Increased muscle tone and contracture late after ischemic stroke. Brain Behav. (2020) 10:807–17. doi: 10.1002/brb3.1509, 31893564 PMC7010575

[79] Meseguer-Henarejos A-B Sanchez-Meca J Lopez-Pina J-A Carles-Hernandez R. Inter- and intra-rater reliability of the modified Ashworth scale: a systematic review and meta-analysis. Eur J Phys Rehabil Med. (2018) 54:1019–25. doi: 10.23736/s1973-9087.17.04796-7, 28901119

[80] Zhu T Liu K Ni B-Y Li L Jin H-P Wu W. Effects of extracorporeal shock wave therapy combined with isokinetic strength training on spastic calf triceps in patients after a stroke: a double-blinded randomised controlled trial. Neurol Res. (2023) 45:1019–25. doi: 10.1080/01616412.2023.2255413, 37668321

[81] Opheim A Danielsson A Alt Murphy M Persson HC Sunnerhagen KS. Upper-limb spasticity during the first year after stroke. Am J Phys Med Rehabil. (2014) 93:884–96. doi: 10.1097/phm.0000000000000157, 25180838

[82] Liu Z Li Z Duan C. Effects of Maitland mobilization technique on upper extremity function in stroke survivors with spasticity: an experimental study. Medicine (Baltimore). (2024) 103:e38184–9. doi: 10.1097/md.0000000000038184 38758885 PMC11098168

[83] Lee S. The effects of exercise with TENS on spasticity, balance, and gait in patients with chronic stroke: a randomized controlled trial. Med Sci Monit. (2014) 20:1890–6. doi: 10.12659/msm.890926, 25300431 PMC4206395

[84] Wang Y-C Chang P-F Chen Y-M Lee Y-C Huang S-L Chen M-H. Comparison of responsiveness of the Barthel index and modified Barthel index in patients with stroke. Disabil Rehabil. (2022) 45:1097–102. doi: 10.1080/09638288.2022.2055166, 35357990

[85] Kim S-Y Lee M-Y Lee B-H. Effects of rehabilitation robot training on physical function, functional recovery, and daily living activities in patients with sub-acute stroke. Medicina (Kaunas). (2024) 60:811–26. doi: 10.3390/medicina60050811, 38792996 PMC11123305

[86] Park H-K Lee H-J Lee S-J Lee W-H. Land-based and aquatic trunk exercise program improve trunk control, balance and activities of daily living ability in stroke: a randomized clinical trial. Eur J Phys Rehabil Med. (2020) 55:687–94. doi: 10.23736/s1973-9087.18.05369-8, 30370752

