# Supplementary material for: A systematic review and meta-analysis of the effects of exercise training on dysfunction in acute, subacute, and chronic stroke patients
Source: Front Neurol. 2026 May 14;17:1740742. doi: 10.3389/fneur.2026.1740742 (PMC13215809; doi:10.3389/fneur.2026.1740742)
Supplement: Supplementary file 1 [file Table_1.docx]

**Supplemental table 1. Search strategy for identification of studies to be included in the review**

A systematic review and meta-analysis of the effects of early exercise training on dysfunction in stroke patients

| **Search strategy**  #1 ("Exercise"[Mesh] OR "Exercise*"[Title/Abstract] OR "Physical Activity*"[Title/Abstract] OR "Physical Exercise*"[Title/Abstract] OR "Isometric Exercise*"[Title/Abstract] OR "Aerobic Exercise*"[Title/Abstract] OR" Exercise Training*"[Title/Abstract] OR "Training"[Title/Abstract])  #2 ("Acute Stage"[Title/Abstract] OR "Acute Phase"[Title/Abstract] OR"Acute Period"[Title/Abstract] OR"Acute Time"[Title/Abstract] OR"Early Stage"[Title/Abstract] OR"Early Phase"[Title/Abstract])  #3 ("Subacute Stage"[Title/Abstract] OR "Subacute Phase"[Title/Abstract] OR"Subacute Period"[Title/Abstract] OR"Subacute Time"[Title/Abstract])  #4 ("Chronic Stage"[Title/Abstract] OR "Chronic Phase"[Title/Abstract] OR"Chronic Period"[Title/Abstract] OR"Chronic Time"[Title/Abstract]OR "Convalescence*"[Title/Abstract] OR"Recovery Phase"[Title/Abstract]OR"Convalescent Period"[Title/Abstract] OR"Convalescent stage"[Title/Abstract] OR"decubation"[Title/Abstract] OR"Restoration Stage"[Title/Abstract] OR"Sequela Period"[Title/Abstract] OR"Sequel Stage"[Title/Abstract])  #5 ("Stroke"[Mesh] OR "Stroke*"[Title/Abstract] OR "Cerebrovascular Accident*"[Title/Abstract] OR "Cerebrovascular Apoplexy"[Title/Abstract] OR "Brain Vascular Accident*"[Title/Abstract] OR "Apoplexy"[Title/Abstract] OR "Cerebral Infarct*"[Title/Abstract] OR "Subcortical Infarction*"[Title/Abstract] OR "Posterior Choroidal Artery Infarction"[Title/Abstract] OR "Anterior Choroidal Artery Infarction"[Title/Abstract] OR "Infarction of Brain"[Title/Abstract] OR "Brain Infarction"[Title/Abstract] OR "Cerebral Haemorrhage"[Title/Abstract] OR "Cerebrum Hemorrhage*"[Title/Abstract] OR "Cerebral Parenchymal Hemorrhage*"[Title/Abstract] OR "Intracerebral Hemorrhage*"[Title/Abstract] OR "Hematencephalon"[Title/Abstract] OR "Encephalorrhagia"[Title/Abstract] OR "Brain Haemorrhage"[Title/Abstract])  #6 (#1 AND #2 AND #3 AND #4 AND #5 ) |
| --- |

1)878 articles were identified: PubMed-106, Cochrane-375, Embase-153, Web of science-244

2) 651 relevant citations screened after duplicates removed

3)507 relevant citations screened by automation tools

4)278 relevant citations screened by title and abstract

5) 6 articles were included for meta-analysis after removing 271 articles with reasons by reading full-text.
